# Supplementary material for: Deciphering Machine Learning Decisions to Distinguish between Posterior Fossa Tumor Types Using MRI Features: What Do the Data Tell Us?
Source: Cancers (Basel). 2023 Aug 8;15(16):4015. doi: 10.3390/cancers15164015 (PMC10452543; doi:10.3390/cancers15164015)
Supplement: Supplementary file 1 [file cancers-15-04015-s001.zip › cancers-2496390-supplementary.pdf]

S1: Additional Figures

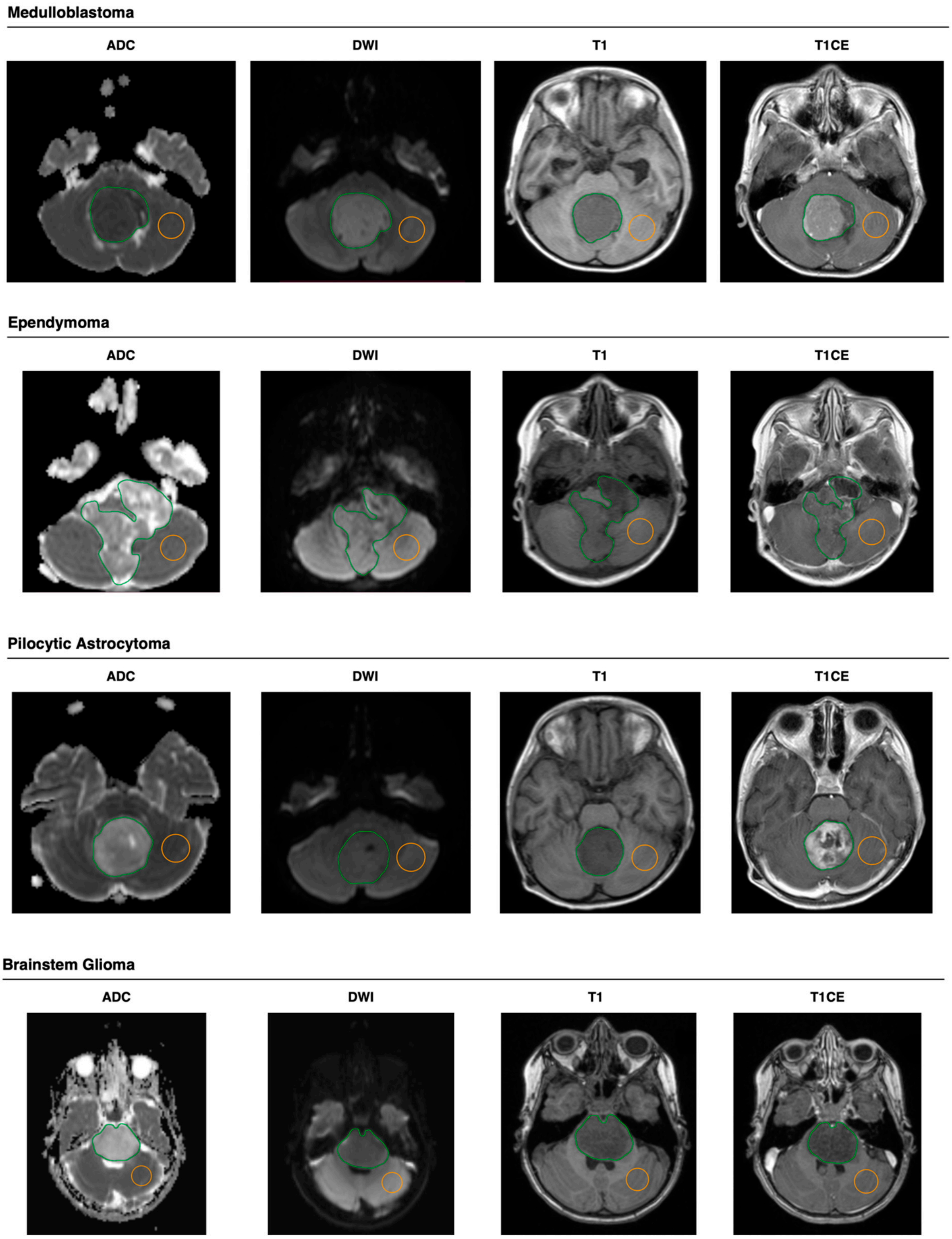

Figure S1: Examples of ROI delineations on a ADC, DWI, T1 and T1CE MRI.

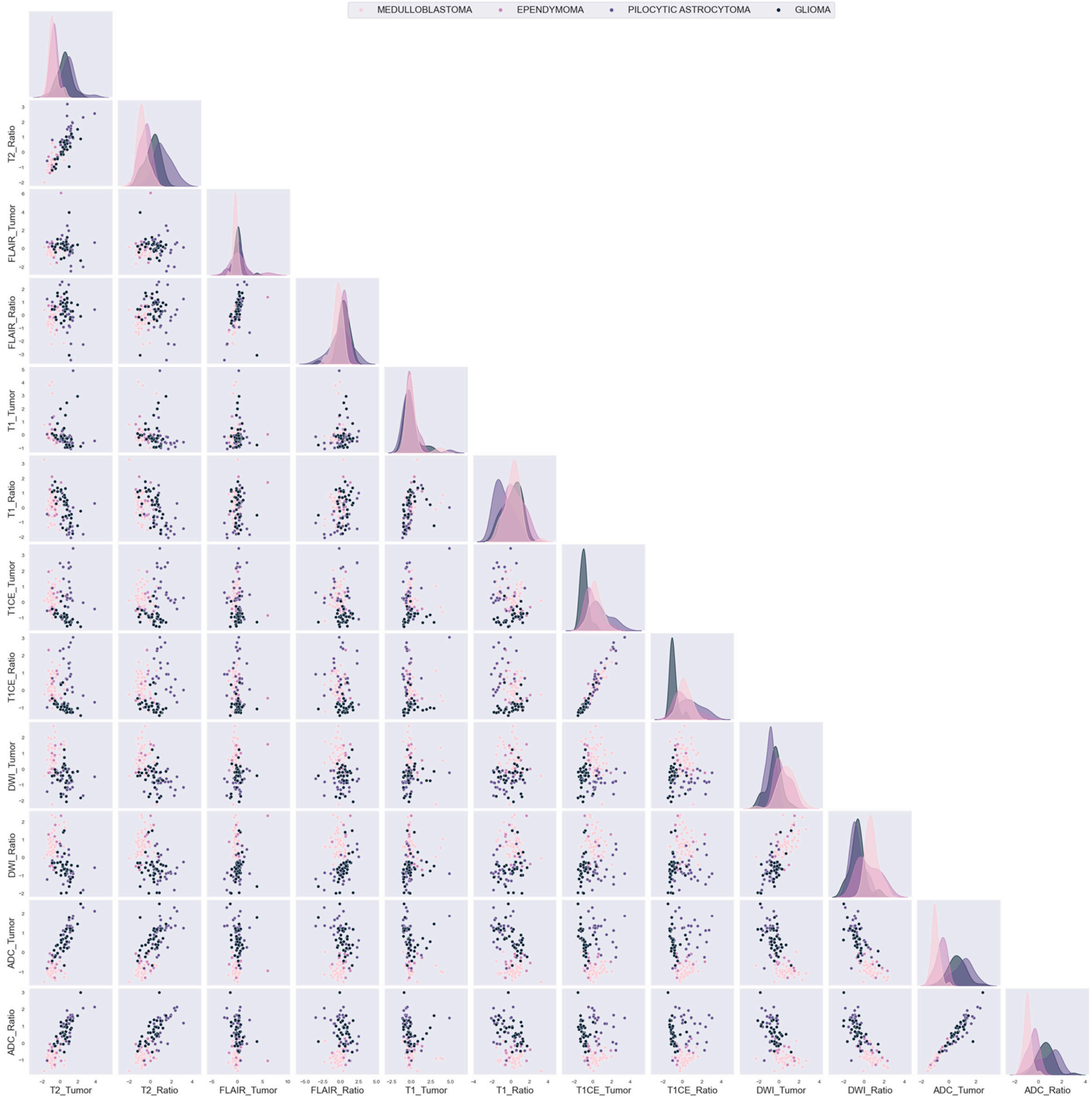

Figure S2: Pairwise plot with no parenchymas.

States	Precision	Recall	Accuracy	F1 Score
State 1	80.32	73.74	82.35	75.80
State 42	77.71	72.07	82.35	72.99
State 123	86.51	72.21	82.35	72.22
State 1234	78.95	73.49	82.35	75.22
State 12345	60.35	65.16	78.43	62.43
Averaged	76.77	71.34	81.57	71.74

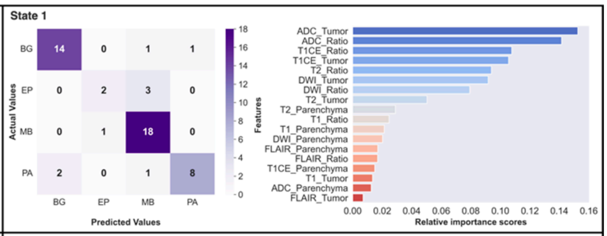

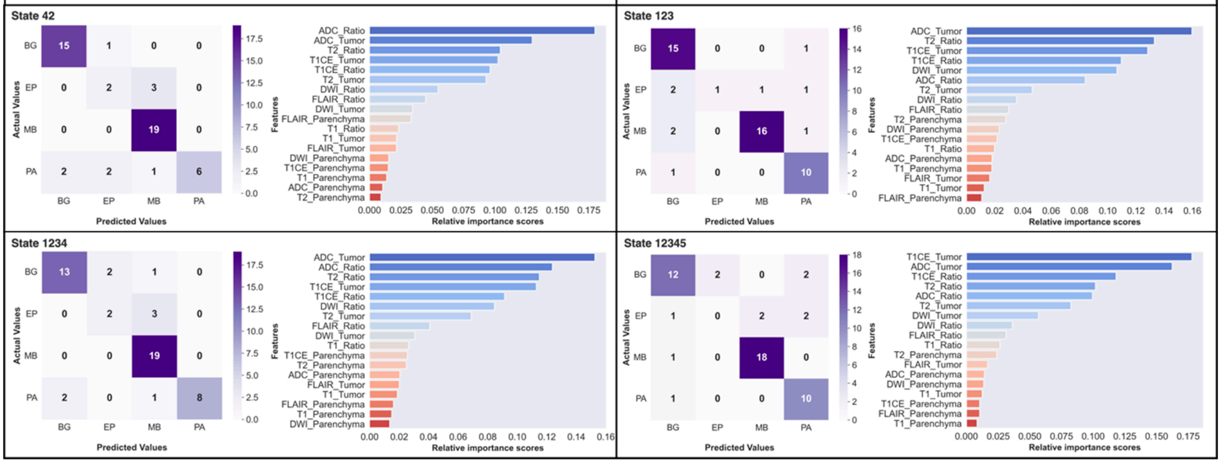

Figure S3: RF model analysis of MB, EP, PA, and BG with classification metrics, confusion matrices, and feature importance distributions for each stratified random sampling state.

High quality pdf version of figures can be found at our GitHub repository.

S2: Differentiation scores of machine learning models for all tumor types.

Table S1. MB-EP

Models	Precision	Recall	Accuracy	F1_Score
**CB**	70.62 ± 28.33	56.00 ± 5.48	81.67 ± 2.28	54.82 ± 9.70
**DT**	67.36 ± 11.68	68.42 ± 13.09	73.33 ± 14.01	65.63 ± 12.70
**GBM**	64.99 ± 21.04	62.63 ± 14.72	75.83 ± 9.03	61.57 ± 15.21
**LR**	70.65 ± 4.55	68.21 ± 7.86	80.00 ± 3.49	67.70 ± 6.19
**LSVM**	65.93 ± 11.66	68.00 ± 13.76	75.00 ± 8.84	65.33 ± 11.55
**RF**	83.58 ± 12.02	62.95 ± 5.43	83.33 ± 2.95	65.38 ± 6.86
**SVM**	67.48 ± 11.11	69.05 ± 11.95	76.67 ± 6.97	66.47 ± 10.16
**XGB**	70.36 ± 24.08	58.84 ± 10.52	79.17 ± 7.80	60.08 ± 13.52

Table S2. MB-PA

Models	Precision	Recall	Accuracy	F1_Score
**CB**	94.67 ± 4.51	94.56 ± 5.94	94.84 ± 4.89	94.48 ± 5.36
**DT**	92.01 ± 3.19	92.46 ± 4.68	92.26 ± 3.68	91.86 ± 4.04
**GBM**	92.59 ± 2.98	92.98 ± 4.78	92.90 ± 3.53	92.50 ± 3.96
**LR**	96.60 ± 2.47	95.61 ± 4.78	96.13 ± 3.53	95.81 ± 3.98
**LSVM**	97.46 ± 1.57	97.28 ± 1.70	97.42 ± 1.44	97.28 ± 1.52
**RF**	93.90 ± 3.61	94.04 ± 5.43	94.19 ± 4.21	93.81 ± 4.66
**SVM**	94.96 ± 3.07	94.25 ± 3.28	94.84 ± 2.89	94.51 ± 3.10
**XGB**	91.37 ± 5.64	89.56 ± 7.47	90.97 ± 6.20	90.15 ± 6.91

Table S3. MB-BG

Models	Precision	Recall	Accuracy	F1_Score
**CB**	94.04 ± 3.61	93.42 ± 3.85	93.71 ± 3.73	93.62 ± 3.81
**DT**	93.59 ± 3.19	92.80 ± 3.37	93.14 ± 3.26	93.03 ± 3.33
**GBM**	94.04 ± 3.61	93.42 ± 3.85	93.71 ± 3.73	93.62 ± 3.81
**LR**	94.94 ± 2.38	94.77 ± 2.50	94.86 ± 2.39	94.81 ± 2.42
**LSVM**	94.47 ± 3.27	94.14 ± 3.73	94.29 ± 3.50	94.21 ± 3.59
**RF**	94.04 ± 3.61	93.42 ± 3.85	93.71 ± 3.73	93.62 ± 3.81
**SVM**	93.39 ± 3.09	92.89 ± 3.42	93.14 ± 3.26	93.05 ± 3.34
**XGB**	94.04 ± 3.61	93.42 ± 3.85	93.71 ± 3.73	93.62 ± 3.81

Table S4. EP-PA

Models	Precision	Recall	Accuracy	F1_Score
**CB**	91.45 ± 8.40	93.00 ± 6.47	91.76 ± 8.92	90.94 ± 9.04
**DT**	84.64 ± 9.39	89.17 ± 7.57	84.71 ± 10.69	83.99 ± 10.85
**GBM**	84.64 ± 9.39	89.17 ± 7.57	84.71 ± 10.69	83.99 ± 10.85
**LR**	95.90 ± 4.17	96.33 ± 4.11	96.47 ± 3.22	95.80 ± 3.85
**LSVM**	88.60 ± 7.09	91.33 ± 5.16	89.41 ± 7.67	88.37 ± 7.53
**RF**	92.62 ± 8.64	93.83 ± 5.82	92.94 ± 7.67	92.18 ± 8.15
**SVM**	90.98 ± 7.58	91.83 ± 5.93	91.76 ± 6.71	90.58 ± 7.21
**XGB**	83.81 ± 9.69	86.33 ± 7.21	82.35 ± 11.76	81.48 ± 11.35

Table S5. EP-BG

Models	Precision	Recall	Accuracy	F1_Score
**CB**	76.68 ± 11.87	73.75 ± 9.51	80.95 ± 6.73	73.34 ± 8.76
**DT**	76.88 ± 12.74	77.12 ± 12.45	81.90 ± 8.52	75.56 ± 12.02
**GBM**	77.21 ± 12.86	75.75 ± 11.90	81.90 ± 8.52	75.02 ± 11.74
**LR**	91.46 ± 7.58	87.50 ± 10.94	91.43 ± 6.21	87.48 ± 9.73
**LSVM**	84.88 ± 9.44	87.00 ± 12.25	88.57 ± 7.22	84.51 ± 11.02
**RF**	71.62 ± 21.29	71.62 ± 14.83	81.90 ± 6.21	69.96 ± 16.64
**SVM**	85.82 ± 5.24	84.25 ± 8.77	88.57 ± 2.61	83.76 ± 4.79
**XGB**	76.68 ± 11.87	73.75 ± 9.51	80.95 ± 6.73	73.34 ± 8.76

Table S6. PA-BG

Models	Precision	Recall	Accuracy	F1_Score
**CB**	89.95 ± 5.97	88.69 ± 6.86	89.63 ± 6.09	89.04 ± 6.50
**DT**	81.00 ± 5.36	78.92 ± 5.12	80.74 ± 4.83	79.48 ± 5.12
**GBM**	79.45 ± 6.44	77.67 ± 5.50	79.26 ± 5.62	78.08 ± 5.67
**LR**	84.07 ± 3.44	84.32 ± 2.77	84.44 ± 3.10	84.02 ± 3.05
**LSVM**	82.75 ± 3.54	82.50 ± 2.62	82.96 ± 3.31	82.40 ± 3.12
**RF**	89.38 ± 6.26	86.59 ± 7.56	88.15 ± 6.63	87.25 ± 7.15
**SVM**	84.40 ± 4.28	83.75 ± 3.42	84.44 ± 4.06	83.85 ± 3.92
**XGB**	90.41 ± 5.04	88.41 ± 5.24	89.63 ± 4.83	89.01 ± 5.19

Table S7. MB-EP-PA-BG

Models	Precision	Recall	Accuracy	F1_Score
**CB**	72.60 ± 9.38	68.82 ± 4.65	79.22 ± 2.97	69.31 ± 5.93
**DT**	70.54 ± 8.88	67.41 ± 4.85	76.86 ± 3.77	67.90 ± 6.37
**GBM**	62.98 ± 5.09	60.60 ± 2.59	71.76 ± 3.28	60.86 ± 2.99
**LR**	74.21 ± 11.20	67.98 ± 3.74	79.22 ± 3.28	68.03 ± 4.74
**LSVM**	66.61 ± 9.53	65.68 ± 2.74	77.25 ± 1.75	64.56 ± 4.14
**RF**	76.77 ± 9.78	71.34 ± 3.53	81.57 ± 1.75	71.74 ± 5.41
**SVM**	70.05 ± 6.38	64.94 ± 2.38	74.51 ± 2.77	65.21 ± 2.26
**XGB**	69.45 ± 3.09	67.29 ± 2.94	76.47 ± 2.40	67.28 ± 3.13
